# FOCUS4 biomarker laboratories: from the benefits to the practical and logistical issues faced during 6 years of centralised testing

**DOI:** 10.1136/jclinpath-2022-208233

**Published:** 2022-03-07

**Authors:** Susan D Richman, Gemma Hemmings, Helen Roberts, Niall Gallop, Rachel Dodds, Lyndsay Wilkinson, Jonathan Davis, Rhian White, Emma Yates, Bharat Jasani, Louise Brown, Tim S Maughan, Rachel Butler, Philip Quirke, Richard Adams

**Affiliations:** 1 Leeds Institute on Medical Research, University of Leeds, Leeds, UK; 2 All Wales Molecular Genetics Laboratory, All Wales Medical Genetics Service, University Hospital of Wales, Cardiff, UK; 3 MRC Clinical Trials Unit at UCL, London, UK; 4 TARGOS Molecular Pathology GmbH, Kassel, Germany; 5 MRC Oxford Institute for Radiation Oncology, University of Oxford, Oxford, UK; 6 Velindre Cancer Centre, Cardiff University, Cardiff, UK

## Abstract

**Aims:**

FOCUS4 was a phase II/III umbrella trial, recruiting patients with advanced or metastatic colorectal cancer, between 2014 and 2020. Molecular profiling of patients’ formalin-fixed, paraffin-embedded tumour blocks was undertaken at two centralised biomarker laboratories (Leeds and Cardiff), and the results fed directly to the Medical Research Council Clinical Trials Unit, and used for subsequent randomisation. Here the laboratories discuss their experiences.

**Methods:**

Following successful tumour content assessment, blocks were sectioned for DNA extraction and immunohistochemistry (IHC). Pyrosequencing was initially used to determine tumour mutation status (KRAS, NRAS, BRAF and PIK3CA), then from 2018 onwards, next-generation sequencing was employed to allow the inclusion of TP53. Protein expression of MLH1, MSH2, MSH6, PMS2 and pTEN was determined by IHC. An interlaboratory comparison programme was initiated, allowing sample exchanges, to ensure continued assay robustness.

**Results:**

1291 tumour samples were successfully analysed. Assay failure rates were very low; 1.9%–3.3% for DNA sequencing and 0.9%–1.3% for IHC. Concordance rates of ≥98% were seen for the interlaboratory comparisons, where a result was obtained by both laboratories.

**Conclusions:**

Practical and logistical problems were identified, including poor sample quality and difficulties with sample anonymisation. The often last-minute receipt of a sample for testing and a lack of integration with National Health Service mutation analysis services were challenging. The laboratories benefitted from both pretrial validations and interlaboratory comparisons, resulting in robust assay development and provided confidence during the implementation of new sequencing technologies. We conclude that our centralised approach to biomarker testing in FOCUS4 was effective and successful.

## Introduction

**Figure 1:**
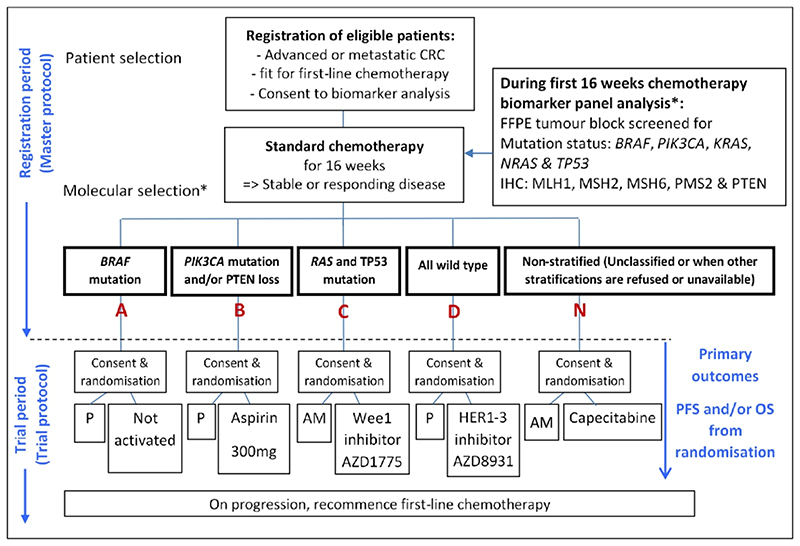
FOCUS4 trial schema. *The molecular cohorts shown here are in a molecular hierarchical order, from left to right. AM, active monitoring; CRC, colorectal cancer; FFPE, formalin-fixed, paraffin-embedded; IHC, immunohistochemistry; P, placebo; PFS, progression-free survival; OS, overall survival.

We are seeing an increase in clinical trials, requiring biomarker assessment to randomise patients to a particular treatment arm or drug regimen. FOCUS4 followed several trials for patients with colorectal cancer (CRC), such as PICCOLO,^1 2^ FOCUS3 (3) and FOxTROT, where this was required. The uniqueness of FOCUS4 lay in its groundbreaking, umbrella trial design, which when it opened in 2014, was one of the first molecularly stratified platform trials in the world.^3^ The multiarm, multistage trial design, allowed several biological cohorts to run in parallel, with each having its own control arm, following the molecular stratification (see figure 1). The adaptive nature of FOCUS4 used predefined and preplanned interim analysis points, to determine whether a particular treatment was showing a sufficiently strong signal to justify keeping the cohort open.

Patients with *KRAS*, *NRAS*, *PIK3CA* and *BRAF* wild-type tumours were randomised between the pan-HER inhibitor AZD8931 and placebo. Following the first planned interim analyses, the Independent Data Monitoring Committee and the Trial Management Group (TMG), closed the FOCUS4-D cohort and reported the results.^4^ FOCUS4-B closed early, as it failed to recruit sufficient patient numbers. FOCUS4-N accepted patients whose biomarker results were inconclusive or unavailable, patients who did not wish to enter a molecular cohort, or where no suitable molecular cohort was open. Patients were randomised between capecitabine and active monitoring, with the results providing additional evidence supporting patients being offered treatment breaks, following first-line therapy.^5^ FOCUS4-A was never activated, due to a lack of pharmaceutical company interest. The results of FOCUS4-C, where patients whose tumours were both *RAS*-mutant and *TP53*-mutant, were randomised between adavosertib and active monitoring, showed that adavosertib improved progression free survival), and importantly for the patients, was well tolerated.^6^

All FOCUS4 samples were processed by two centralised laboratories. The Leeds laboratory, (Leeds Institute of Medical Research) and the laboratories in Cardiff, (Department of Pathology and All Wales Medical Genomics Service, University Hospital of Wales), had previously worked jointly to deliver the biomarker testing on the FOCUS3 trial.^7^ Before commencing FOCUS3, the laboratories undertook a prestudy interlaboratory sample exchange, demonstrating 100% concordance. This quality assurance programme for sample exchange and blinded mutation screening was developed further, prior to FOCUS4 opening to recruitment, to include immunohistochemistry (IHC). Ninety-seven metastatic CRC (mCRC) samples were processed in both laboratories, according to FOCUS4 protocols, ensuring processing pipelines were optimised, and pyrosequencing and IHC in both laboratories would yield concordant results. Two samples (2.1%) gave discrepant pyrosequencing results, likely due to tumour heterogeneity, as the laboratories used different sections of each block for DNA extraction. The few pTEN IHC discrepancies and mismatch repair (MMR) IHC discrepancies were resolved following joint review.^8^

Laboratory teams are often the forgotten stakeholder, in terms of the rollout and running of a multi-national clinical trial. Throughout FOCUS4, the laboratories worked together to provide interlaboratory comparison data and constructive feedback to the Medical Research Council Clinical Trials Unit (MRCCTU) and provided an insightful viewpoint to monthly TMG meetings.

Here, we present the results of the joint laboratory analyses and interlaboratory comparisons and discuss the benefits of centralised testing, and the practical and logistical issues encountered during FOCUS4.

## Materials And Methods

The trial recruited participants until March 2020, when it was closed because of the COVID-19 pandemic, just before its scheduled closure date of July 2020. Follow-up continued until October 2020 and results were reported elsewhere.^4–6^

### Participants

Patients were eligible for trial registration, if aged ≥18, and presenting with newly diagnosed, mCRC. 103 hospitals opened to recruitment across the UK, with 88 registering at least one patient. During 16 weeks of induction chemotherapy, eligible patients were registered and a representative formalin-fixed, paraffin-embedded (FFPE) tumour block retrieved from Histopathology, and forwarded to one of the centralised testing laboratories. All patients provided informed consent, for biomarker testing on their sample.

### FFPE tumour sample processing-1

Tumour blocks were sectioned, with the top section being H&E-stained, using standard laboratory procedures. Additional sections were taken for DNA extraction and IHC. Each H&E was reviewed, to confirm the presence of sufficient tumour tissue, and an area for macro-dissection was highlighted.

#### Pyrosequencing

DNA extraction was carried out in Leeds using the QIAamp DNA extraction Kit, and in Cardiff using the EZ1 DNA tissue kit (Qiagen, Manchester, UK), according to the manufacturer’s instructions. Pyrosequencing was undertaken using the Pyro-Mark Q96 (Qiagen, Manchester, UK), analysing mutation hotspots within *KRAS* codons 12, 13, 61 and 146; *NRAS* codons 12, 13 and 61; *BRAF* codon 600 and *PIK3CA* codons 542, 545–6 and 1047. Appropriate positive and negative controls were included in each run. The programmes were analysed by trained personnel, and results uploaded directly to the FOCUS4 trial MACRO database.

#### Immunohistochemistry

Five markers were assessed by IHC, on a DAKO Autostainer Link 48 (DAKO, Ely, UK), using preprogrammed protocols. Ready-to-use antibodies (IR079, IR085 and IR086) were used to assess MLH1, MSH2 and MSH6, respectively. DAKO PMS2 (M3674) and pTEN (M3627) were used at predetermined dilutions (1/40 and 1/100, respectively). Tumours were deemed proficient MMR, if the tumour nuclei stained positively for MLH1, MSH2, MSH6 and PMS2. If all the tumour nuclei were negative for one or two of these proteins, the tumour was classified as deficient MMR. As a positive, internal control, evidence of staining in stromal cells and infiltrating lymphocytes was required. Constitutive pTEN staining was expected in the tumour cytoplasm. Each tumour was classed as either ‘positive’, where there was retention of staining or ‘negative’ where there was no evidence of staining. Example images can be seen elsewhere.^8^ Results were uploaded directly to the FOCUS4 trial MACRO database.

### FFPE tumour sample processing-2

From 2018 onwards, an amended processing pipeline was implemented, due to the opening of the FOCUS4-C randomisation.^6^ Pyrosequencing was unsuitable for assessing the mutational status of *TP53*, so next-generation sequencing (NGS) was employed. In advance of this technology shift, interlaboratory validations were undertaken, with the results being presented here.

Due to the low weekly recruitment numbers, (n<10), it was deemed cost-ineffective to continue running the NGS platform in Leeds. Furthermore, the Cardiff National Health Service Histopathology laboratory could no longer support the demands of the trial, so all sequencing analysis was undertaken in Cardiff, and all IHC was undertaken in Leeds, as previously described. FFPE blocks continued to be sent to their originally allocated biomarker laboratory. Blocks arriving in Cardiff, were forwarded to Leeds for sectioning and subsequent H&E assessment. The annotated H&E section, plus unstained sections were shipped to Cardiff, for DNA extraction and NGS. During this period, where NGS was performed in a single laboratory, Cardiff participated in appropriate External Quality Assurance schemes. On trial closure, all FFPE tumour blocks were transferred to the Wales Cancer Bank for long-term storage, under their own ethics.

#### Next-generation sequencing

The GeneRead Clinically Relevant Mutation panel (Qiagen, Manchester), interrogates a panel of 24 genes. GeneReadDNA Targeted Panels V2 was used, according to the manufacturer’s instructions. A bioinformatics pipeline was designed to determine the mutation status of each tumour sample for *KRAS*, *NRAS*, *BRAF*, *PIK3CA* and *TP53*. This filtered known polymorphisms and sequencing artefacts; any remaining variants present at ≥5% allele frequency were viewed in the Integrated Genomics Viewer (https://igv.org). The actionability of variants was based on FOCUS4 guidelines, with variant investigations involving review in databases such as COSMIC (https://cancer.sanger.ac.uk/cosmic), literature review, and the use of protein prediction software performed as necessary to determine the actionability of variants. Registered Clinical Scientists assessed all variants, and results uploaded directly to the FOCUS4 trial MACRO database.

### Interlaboratory exchanges

For the duration of the trial, the laboratories undertook interlaboratory exchanges, twice each year, where samples were swapped between the two laboratories, to allow retrospective sequencing in both, and the resultant sequencing data compared. Initially only pyrosequencing was used, but from August 2016, NGS was also incorporated as both laboratories were moving to this platform.

### Lessons learned

Following the trial closure, the biomarker teams had the opportunity to reflect on their experiences, as one of the Trial stakeholders.^9^ Here we discuss the sample processing pipeline successes, and identify issues which TMGs ought to take into consideration at the early planning stages of future clinical trials.

## Results

### Sample processing

Between January 2014 and March 2020, 1434 patients were registered, and FFPE tumour blocks from 1402 patients sent to either of the centralised laboratories. Four samples were lost in the post, and of the 1398 FFPE blocks received, 581 were resections, and the remaining 817 were biopsies. Almost 80 FFPE blocks contained insufficient tumour material for profiling. 1291 tumour samples underwent successful molecular profiling (defined as sequencing, by either pyrosequencing or NGS, plus IHC), comprising 569 resections and 722 biopsies.

### Sequencing results

The sequencing data are summarised in table 1. Mutation rates for each gene were as expected. Most samples yielded a result, as highlighted by the low assay failure rates; 2.2% for *BRAF*; 1.9% for *KRAS*; 1.9% for *NRAS*; 3.3% for *PIK3CA* and 2.6% for *TP53*. Missing data were recorded for only one sample, with the exception of *TP53*, which was only added to the sequencing panel when FOCUS4-C was opened, by which time, a large number of samples had already been processed, without *TP53* sequencing.

### IHC results

Each tumour was assessed for the expression of pTEN, MLH1, MSH2, MSH6 and PMS2, (table 2). 90.5% of the assessed tumours retained expression of pTEN, with only 7.2% displaying complete loss of expression. As expected for this cohort of aCRC patients, only 2.7% of tumours displayed loss of expression of one or two MMR proteins. Again, very low assay failures rates were observed, with between 0.9% and 1.3% of tumours failing to pass stringent quality controls. These included insufficient tumour material on the slide to allow assessment, either due to cutting through the tumour in the block, or the tissue failing to adhere adequately to the slide during staining. On very rare occasions, the slide failed to stain on the Autostainer.

### Results of interlaboratory comparisons

Sample-swap 1 (May 2015) involved both laboratories sequencing 31 tumour samples. Each was subjected to eight individual assays; *KRAS* codons 12 and 13, 61 and 146; *NRAS* codons 12&13 and 61; *BRAF* codon 600 and *PIK3CA* exons 9 and 20, totaling 248 separate results. 244/248 (98%) were concordant between the two laboratories. The discrepancies were jointly reviewed, and shown to be due to low-level variants, which were missed in one of the laboratories.

Sample-swap 2 (September 2015) involved swapping three samples, with 23 of the 24 separate assays (96%) being concordant. Joint review resolved the discrepancy.

Sample-swap 3 (March 2016) involved swapping six samples. 46 of the 48 separate assays (98%) were concordant. One discrepancy was seen in the naming convention of a complex mutation in *KRAS* codon 12&13 (c.34_35delinsTT in one laboratory, and ‘atypical’ in the other), and one discrepancy was seen in *PIK3CA* exon *9* (it was only detected in one laboratory). It is worth noting that not the same DNA aliquot was used in each laboratory, as each laboratory sectioned and processed the block, as per FOCUS4 protocols.

Sample-swap 4 (August 2016) involved swapping six samples. The three sent from Cardiff were initially assessed there by pyrosequencing, then validated by both pyrosequencing and NGS in Leeds. The three samples sent from Leeds were assessed initially by pyrosequencing, then analysed by both pyrosequencing and NGS in Cardiff. 100% concordance was seen (see table 3).

Sample-swap *5* (May 2017) involved swapping ten samples. Each laboratory provided five samples, which had undergone both pyrosequencing and NGS. The results were validated using NGS at the receiving laboratory. For the five samples sent from Cardiff to Leeds, there was 100% concordance between all three results. Of the samples sent from Leeds to Cardiff, and which were successfully sequenced, there was 100% concordance between platforms and laboratories. Variant allele frequencies were very similar between laboratories (see table 4). The two samples reported as ‘failed’ on NGS, did so because of low sequencing coverage.

## Discussion

During the FOCUS4 trial, each laboratory received, processed and reported results for several hundred samples. Working closely together prior to the first patient entering the trial, the laboratories were able to optimise all assays. These optimisations were critical to the smooth running of the centralised testing strategy that FOCUS4 employed. The close working relationship between laboratories continued throughout the trial, with interlaboratory sample swaps ensuring ongoing quality assurance of assay protocols. Each laboratory communicated directly with Data and Trial Managers at the MRCCTU, enabling real-time sample tracking. Individuals from each laboratory sat on the TMG, which facilitated direct communication regarding any issues, as and when they arose.

Both laboratories encountered the issue of poor sample quality. Almost 80 tumour blocks contained insufficient tumour tissue for processing. It is likely that Histopathology departments receiving block requests simply forwarded them to the biomarker laboratories, without adequate Pathology review. Often the accompanying Pathology report provided details of local sequencing and IHC, which had depleted the tissue, but this was not identified at the time of the request. Each block, still had to be booked in at each laboratory, resulting in wasted technician-time and the necessary request for additional material caused delays in reporting the results. We strongly recommend that a Pathology review is implemented, to ensure sufficient tumour material remains in each block included in a trial,^10^ particularly where local testing has been undertaken. Towards the end of the trial, a larger number of Trusts were carrying out their own sequencing, or having it outsourced, as part of local patient treatment pathways. When FOCUS4 opened in 2014, local testing was in its infancy, hence the use of centralised, cross-validated biomarker laboratories. Although this position altered over the following 6 years, the results of local biomarker screening were not accepted, as the local testing could not be taken through vigorous validation processes. It should be noted that there were no discrepancies between the on-trial results and those obtained through local standard of care pathways.

There were often lengthy delays between the block request date, and the sample arriving in either the Leeds or Cardiff laboratories. The biomarker results had to be reported to the MRCCTU promptly, as once patients completed their 16 weeks of chemotherapy, and had their CT-scan, there was a finite period whereby they could be randomised to one of the molecular comparisons.

A few Trusts were reluctant to release their patients’ tumour blocks, even though patients had consented. These were local policy decisions, often where only the diagnostic tumour block was stored. To circumvent this, these sites sent mounted sections to the laboratories. This did however mean that the sections were not optimally prepared for IHC, but it did allow DNA extraction and subsequent mutation screening to occur.

Minor issues were identified with the completion of the Biomarker case report form (CRF). Patient-identifiable information had to be removed from all paperwork, but this was not always undertaken satisfactorily. On occasion, it was unclear from the CRF whether the patient had consented for their sample to be used in future research. Although only minor issues, these resulted in additional, and unnecessary administration for each biomarker laboratory and the MRCCTU Data Managers.

None of the issues highlighted above are specific to FOCUS4. They were previously identified in 2017, during the Medical Research Council Hubs for Trials Methodology Research Network’s Stratified Medicine Working Group workshop,^11^ as being pertinent to a number of clinical trials; The National Lung Matrix Trial;^12^ TOPARP;^13^ ATLANTIS^14^ and POETIC,^15^ and therefore must be addressed by future TMGs.

The negative aspects of our centralised testing approach were outweighed by the benefits. Through the pretrial validation and interlaboratory sample swaps, we demonstrated consistent assay robustness, as evidenced by the low assay failure rates. Clinical studies seldom publish assay failure rates, although our two biomarker laboratories undertook *RAS* and *BRAF* testing on the FOCUS3 trial, where an assay failure rate of 3.9% was reported.^7^ This is almost identical to the 4% pyrosequencing failure rate reported in TRIBE,^16^ which was the result of insufficient tissue for testing. Comparing these results with studies such as National Cancer Institute of Canada Clinical Trials group Study BR.21,^17^ which reported successful *KRAS* mutation analysis in 206/230 (89.6%) of NSCLC samples, we are clearly demonstrating a successful optimisation and validation strategy.

FOCUS4-C required the move to NGS, to enable the complete gene sequencing of *TP53*. The flexibility afforded us, in combination with the interlab optimisation and validation, resulted in a smooth transition to the new technology.

The biomarker laboratories provided a unique insight into trial documentation issues. The original Biomarker CRF was a two-sided document. On occasion, it was unclear whether the patient had consented for their tumour sample to be used in future research, as the tick-box (on page 2), remained blank. Without this knowledge, the block could not be cored and added into a tissue microarray (TMA), because if consent was subsequently not given, it is almost impossible to remove individual cores without destroying a TMA. Working with the MRCCTU, the form was redesigned, to a single-page document, resulting in no further ambiguity.

Work is now underway on planned blood-based translational research. Both laboratories are currently optimising cfDNA extraction and subsequent analysis pipelines, to make full use of this valuable sample resource. It is planned that patient clinical data will be stored under ethics with the Stratification in COloRecTal cancer (S:CORT) consortium (https://www.s-cort.org/), making it available to external researchers for further interrogations. Additional in-depth analysis of the FOCUS4-C cohort has already been undertaken through S:CORT, and this will also be made available.

Overall, our centralised approach to biomarker testing was undoubtedly successful. Having a second laboratory to take over testing, if any issues arose, such as equipment failure, or staff sickness in one laboratory, ensured that patients were randomised within the required timeframes. The work undertaken by laboratories, often goes unnoticed, however during FOCUS4, both laboratories were always acknowledged. The processing of multiple assays and reporting of almost 1200 tumour samples was a significant undertaking, and being recognised as an important stakeholder is something that should be replicated in other clinical trials.

## Figures and Tables

**Table 1:** Overall sequencing results, obtained by both laboratories

	*BRAF*	*KRAS*	*NRAS*	*PIK3CA*	*TP53*
Mutation detected	125 (9.7%)	666 (51.6%)	72 (5.6%)	179 (13.9%)	481 (37.3%)
Wildtype	1135 (87.9%)	598 (46.3%)	1192 (92.3%)	1066 (82.6%)	229 (17.7%)
Failed samples	28 (2.2%)	24 (1.9%)	25 (1.9%)	43 (3.3%)	19 (2.6%)
Not tested	2 (0.2%)	2 (0.2%)	1 (0.1%)	2 (0.2%)	1 (0.1%)
Missing data	1 (0.1%)	1 (0.1%)	1 (0.1%)	1 (0.1%)	561 (43.4%)*

The breakdown of sequencing results by gene, and outcome for the 1291 tumour samples that were sequenced in either the Leeds or Cardiff laboratories between January 2014 and March 2020.

* As testing of TP53 mutation status only began in 2017, the 561 samples that had been sequenced prior to this date, were not eligible for TP53 mutation screening, hence the large amount of missing data indicated here.

**Table 2:** The breakdown of the immunohistochemical analyses undertaken

	PTEN	MMR proteins (MLH1, MSH2, MSH6 and PMS2)
Protein(s) expression observed	1169 (90.5%)	1222 (94.6%)
Loss of protein expression	91 (7.2%)	33 (2.7%)
Failed samples	11 (0.9%)	16 (1.3%)
Could not be tested	20 (1.5%)	20 (1.5%)

For each protein, the result was reported as either expression, or loss of expression. samples which could not be tested included, but were not limited to, those which were received in the laboratory following the COVID-19 lockdown of March 2020, and those where a tissue mega-block was received, rather than a standard size FFPE tissue block, which was unsuitable for testing on the Autostainer. FFPE, formalin-fixed, paraffin-embedded; MMR, mismatch repair.

**Table 3:** Summary of the on-trial sample swap between Leeds and Cardiff, run in August 2016

**Sample ID**	**Cardiff pyrosequencing (VAF)**	**Leeds pyrosequencing (VAF)**	**Leeds NGS (VAF)**
Sample 1	KRAS c.35G>T (36%)	KRAS c.35G>T (34%)	KRAS c.35G>T (28%) TP53 c.215C>G (41%)
Sample 2	BRAF c.1799T>A (22%)	BRAF c.1799T>A (29%)	BRAF c.1799T>A (21%) TP53 c.215C>G (72%) TP53 c.796G>C (25%)
Sample 3	BRAF c.1799T>A (15%)	BRAF c.1799T>A (22%)	BRAF c.1799T>A (14%) TP53 c.215C>G (64%) TP53 c.524G>A (16%)
**Sample ID**	**Leeds pyrosequencing (VAF)**	**Cardiff pyrosequencing (VAF)**	**Cardiff NGS (VAF)**
Sample 4	BRAF c.1799T>A (52%)	BRAF c.1799T>A (50%)	BRAF c.1799T>A (50%) TP53 c.844C>T (68%)
Sample 5	KRAS c.35G>A (42%) PIK3CA c.1633G>A (51%)	KRAS c.35G>A (55%) PIK3CA c.1633G>A (41%)	KRAS c.35G>A (36%) PIK3CA c.1633G>A (50%)
Sample 6	KRAS c.436G>A (72%)	KRAS c.436G>A (100%)	KRAS c.436G>A (72%) TP53 c.832C>T (66%)

The *TP53* mutations detected by NGS are outside the scope of the pyrosequencing assay panel, so not detected by the latter assay. NGS, next-generation sequencing; VAF, variant allele frequency.

**Table 4:** Summary of the final on-trial sample swap between Leeds and Cardiff, run in May 2017

Sample ID	Cardiff pyrosequencing (VAF)	Cardiff NGS (VAF)	Leeds NGS (VAF)
Sample 1	BRAF c.1798_1799delGTinsAA (~50%)	BRAF c.1798_1799delGTinsAA (48%)	BRAF c.1798G>A BRAF c.1799T>A (49%)*
Sample 2	BRAF c.1798_1799delGTinsAA (~66%)	BRAF c.1798_1799delGTinsAA (66%)	BRAF c.1798G>A BRAF c.1799T>A (65%)*
Sample 3	KRAS c.35G>A (25%) PIK3CA c.3140A>G (37%)	KRAS c.35G>A (15%) PIK3CA c.3140A>G (22%) TP53 c.215C>G (67%) TP53 c.475G>C (28%)	KRAS c.35G>A (12%) PIK3CA c.3140A>G (17%) TP53 c.215C>G (66%) TP53 c.475G>C (27%)
Sample 4	KRAS c.35G>T (31%)	KRAS c.35G>T (21%)	KRAS c.35G>T (17%)
Sample 5	Pyrosequencing not performed on this sample†	TP53 c.215C>T (99%) TP53 c.380C>T (25%) TP53 c.701A>G (14%) TP53 c.994–1G>T (6%)	TP53 c.215C>T (99%) TP53 c.380C>T (15%) TP53 c.701A>G (29%) TP53 c.994–1G>T (10%)
**Sample ID**	**Leeds pyrosequencing (VAF)**	**Leeds NGS result (VAF)**	**Cardiff NGS result (VAF)**
Sample 6	KRAS c.34G>T (52%) PIK3CA c.1633G>A (48%)	KRAS c.34G>T (39%) PIK3CA c.1633G>A (43%)	NGS failed due to low coverage
Sample 7	KRAS c.35G>A (45%)	KRAS c.35G>A (30%) TP53 c.797G>A (32%)	KRAS c.35G>A (35%) TP53 c.797G>A (41%)
Sample 8	KRAS c.436G>A (30%) PIK3CA c.1634A>C (13%)	NGS failed due to low coverage	NGS failed due to low coverage
Sample 9	KRAS c.35G>A (33%)	KRAS c.35G>A (29%) TP53 c.637C>T (60%) TP53 c.215C>G (100%)	KRAS c.35G>A (22%) TP53 c.637C>T (56%) TP53 c.215C>G (100%)
Sample 10	KRAS c.34G>T (38%)	KRAS c.34G>T (28%) PIK3CA c.363C>T (47%) TP53 c.637C>T (60%)	KRAS c.34G>T (30%) PIK3CA c.363C>T (46%) TP53 c.637C>T (62%)

* These two adjacent mutations can also be called as a single mutation, as was the case in Cardiff.

† No pyrosequencing was undertaken on this sample, as it was not a FOCUS4 patient sample, and local testing in Cardiff had switched to NGS, for routine diagnostic testing. The TP53 mutations detected by NGS are outside the scope of the pyrosequencing assay panel, so not detected by the latter assay.

NGS, next-generation sequencing; VAF, variant allele frequency

## Data Availability

Data are available on reasonable request. The sequencing data analysed during this study are available from the corresponding author on reasonable request.

## Take home messages

▶ Worldwide, many clinical trials are currently recruiting participants, where the results of prospective biomarker assays determine randomisation. The data generated by the trial laboratory is often unpublished, and their valuable experiences overlooked. Here we report on behalf of the two centralised biomarker laboratories, who undertook sample processing throughout the FOCUS4 mCRC clinical trial.
▶ We provide detailed information on not only the biomarker assay results, and our on-trial sample swap quality control procedures, but also highlight both logistical and practical issues, which will act as learning points for future trials. The benefits of centralising the FOCUS4 biomarker testing are also discussed.
▶ In addition to highlighting problems encountered during the trial, by the centralised laboratories, we provide helpful insights and suggestions that we recommend are implemented in future clinical studies.
